# Pulse oximetry at two sensor placement sites in conscious foals

**DOI:** 10.1186/s13028-025-00794-w

**Published:** 2025-01-23

**Authors:** Heini Sofia Rossi, Anna Kristina Mykkänen, Jouni Juho Tapio Junnila, Heli Katariina Hyytiäinen

**Affiliations:** 1 Department of Equine and Small Animal Medicine, Faculty of Veterinary Medicine, University of Helsinki, Viikintie 49, 00014 Helsinki, Finland; 2 EstiMates Oy, Lemminkäisenkatu 14–18, 20520 Turku, Finland

**Keywords:** Arterial blood sample, Equine, Horse, Non-anaesthetised, Oxygenation, Pulse oximeter, Reliability, Saturation, Validity

## Abstract

**Background:**

Pulse oximetry has not been thoroughly evaluated for assessment of oxygenation in conscious foals. Compared with invasive arterial blood sampling, it is a painless and non-invasive method for real-time monitoring of blood oxygen saturation. The aim of this prospective clinical study was to evaluate the usability, validity, and reliability of pulse oximetry at two measuring sites (lip and caudal abdominal skin fold) for blood oxygen saturation measurement in conscious foals with and without respiratory compromise. Thirty-two foals under one month of age were used. Nineteen foals had normal respiratory and cardiovascular function, and 13 had pneumonia. Pulse oximetry with a transmittance sensor was performed in triplicate on each foal’s lip (n = 196 measurements) and/or skin fold (n = 338 measurements), and arterial blood sample was collected. The oxygen saturation values measured by pulse oximetry from the lip and skin fold were compared with each other (n = 58 measurement pairs) and with the calculated arterial oxygen saturation based on arterial blood samples (n = 93 measurement pairs). Furthermore, repeatability of the pulse oximetry measurements was assessed.

**Results:**

Measured blood oxygen saturation was clearly associated with the calculated saturation, but on average (± SD) it was 1.8 (± 3.3) percentage units higher from the lip and 5.7 (± 4.3) percentage units higher from the skin fold than the calculated saturation. In concurrent lip and skin fold measurements within a foal, the skin fold measurements were 2.4 (± 2.4) percentage units higher than the lip measurements. The repeatability of three pulse oximetry saturation measurement results was moderate to good and significantly improved when the measurement furthest from the middle-measured value was excluded. The most deviating measurement was often obtained first. Pulse oximetry in general was well tolerated and easy to perform, but as expected in conscious foals, movement and contact problems generated occasional technical difficulties in some individuals.

**Conclusions:**

In conscious foals, pulse oximetry with a transmittance sensor attached to the lip (but not to the skin fold) is a clinically applicable and valid method for arterial blood oxygen saturation determination. Several measurements should be obtained and outliers discarded to obtain a reliable result.

**Supplementary Information:**

The online version contains supplementary material available at 10.1186/s13028-025-00794-w.

## Background

Hypoxaemia is a common and clinically relevant condition in young foals. It can be linked to physiological adaptation shortly after birth but may also be related to several disease processes such as prematurity, sepsis, neonatal maladjustment syndrome, pneumonia, and other respiratory tract conditions [1, 2]. In young foals, oxygenation is currently assessed primarily by arterial blood gas analysis in clinical practice. The sample is typically obtained from the dorsal metatarsal artery, but technically any palpable artery can be used [1]. However, while the method is objective, there are several disadvantages to arterial blood sampling, including technical difficulties obtaining the sample, pain, bleeding, and haematoma formation, vascular trauma or arteritis following repeated sampling, embolism or thrombosis, poor patient co-operation, and the effect of patient position and age on oxygenation values [3, 4]. A non-invasive alternative for monitoring a foal’s oxygenation is pulse oximetry, but this technique has not been thoroughly tested and evaluated in conscious foals, and reliable readings have been considered challenging to obtain in clinical practice [1]. In human medicine, pulse oximetry is commonly used in health care to estimate the percentage of oxygen saturation of arterial blood haemoglobin (SpO_2_) [5–8], and the method is regularly used also in small animal veterinary medicine, especially during intensive care and procedures requiring anaesthesia [9–14].

Pulse oximeters are electronic medical devices that use the emission of red and infrared light passing through tissues to display a calculated estimate, indicated by SpO_2_, of the true arterial blood oxygen saturation (SaO_2_) [15]. This estimate, obtained by either transmittance or reflectance oximetry, is based on the differing absorption of light by oxyhaemoglobin and deoxyhaemoglobin, and the blood flow through the tissues. Although arterial blood gas analysis is often considered the gold standard for evaluating oxygenation, pulse oximetry offers several practical advantages; it is a non-invasive, easy-to-use, convenient, and cost-effective method for monitoring real-time blood oxygen saturation, even continuously or long-term [6]. As equine practices often do not have access to blood gas analysis especially in the field setting, a reliable and non-invasive, a portable method like pulse oximetry for assessing the patient’s oxygenation could be useful in clinical ambulatory practice. It can also be applicable in a hospital setting, where it could facilitate real-time and rapid decision-making regarding the patient’s treatment. However, the limitations of the method (e.g., inability to measure arterial oxygen or carbon dioxide tension, detect hyperoxemia, or assess ventilation) should be always considered in clinical practice.

To be clinically usable, the validity (level of measurement accuracy) and reliability (level of measurement consistency) of any outcome measurement method need to be assessed. Moreover, repeatability (level of variation in repeated measurements on the same subject under similar conditions) as part of reliability assessment must be evaluated. The use and accuracy of pulse oximeter have been investigated earlier mainly in anaesthetised foals. Pulse oximetry in general appeared to be a useful method to assess arterial oxygen saturation and to detect desaturation in anaesthetised foals, but there were differences in sensor types (transmission or reflectance) and sensor placement sites; some overestimate while others underestimate the arterial oxygen saturation relative to the calculated SaO_2_ [16]. The accuracy of two pulse oximeters with different sensor placements (lip, tongue, ear, rectum) in anaesthetised neonatal foals was assessed, and the conclusion was that the lip and tongue measurements gave the most accurate estimate of SaO_2_ [17]. However, conscious foals have been sparsely evaluated. In one study [18], a reflectance probe at the tail base of conscious neonatal foals provided saturation values of acceptable agreement with calculated SaO_2_, but other sensor placement sites have not been assessed in conscious foals to date.

The aim of this study was to assess the validity and reliability of pulse oximetry at two sensor placement sites (lip and caudal abdominal skin fold) for blood oxygen saturation measurement in conscious foals restrained in lateral recumbency with and without respiratory compromise. Moreover, the aim was to compare the pulse oximetry saturation measurements with the saturation results calculated from the blood gas analysis values, and to compare the two sites for pulse oximetry measurement with each other. The hypothesis was that the pulse oximetry measurements, obtained from both measuring sites and compared with blood gas analysis results, would be valid and have high intrarater reliability.

## Methods

### Animals

A total of 32 client-owned foals (14 fillies, 18 colts) under one month of age were used in this prospective study conducted in a clinical setting with convenience sampling. Foals fulfilling the inclusion criteria that were patients at the University of Helsinki Veterinary Teaching Hospital during 2022–2023 were recruited. The Finnish Project Authorisation Board approved the study (licence number ESAVI/37496/2021). Foal owners provided a signed informed consent prior to participation in the study.

Foals with pneumonia (n = 13) and foals without respiratory compromise (control group, n = 19) were recruited. The pneumonia group included self-ventilating foals aged up to one month that were diagnosed with pneumonia. The pneumonia diagnosis was based on clinical examination, blood sample analyses, compatible findings in chest radiography and/or ultrasonography, and/or hypoxaemia. Foals with pulmonary hypertension, marked cardiovascular instability, cardiac disease, or clinically significant upper airway disease were excluded from the study. The control group included 9 healthy foals up to one month of age that were brought to the hospital accompanying the dam and participated with the owner’s consent, and 6 foals that were admitted to hospital for some reason other than cardiovascular or respiratory disease (e.g., orthopaedic disease, localised infection like umbilical infection, meconium impaction). In addition, four of the recruited foals in the control group were examined at their home stable. The inclusion criterion of the control group was a clinically normal cardiovascular and respiratory status. Six foals with a condition other than cardiovascular or respiratory disease were included in the control group only after the condition potentially affecting the cardiovascular status was resolved and when their cardiovascular and respiratory status were considered normal by an equine specialist. Foals were considered healthy based on the lack of any clinical signs of a disease, physical examination, and in some cases possible blood sample analyses based on the clinician’s assessment. Exclusion criteria were the same as for the pneumonia group, and foals with significant systemic disease like sepsis were also excluded from the control group.

### Study design

On admission to the study, foals underwent clinical examination, including the recording of vital signs (body temperature, heart rate, respiratory rate), and were then restrained in lateral recumbency without sedation by two veterinary nurses holding the limbs or neck as needed. Immediately after this within approximately one minute, an arterial blood sample was anaerobically obtained from the dorsal metatarsal artery for blood gas analysis (Pico 50, Radiometer Medical, Copenhagen, Denmark). The sample was analysed within 5 min of collection (ABL800 Flex, Radiometer Medical or for four foals sampled at home stables Epoc, Epocal Inc., Ottawa, Ontario, Canada). The ABL800 Flex analyser was automatically calibrated daily during the study period with gas standards and calibration solutions provided by the manufacturer, and the handheld Epoc analyser’s calibration was performed with Epoc test cards provided by the manufacturer. Calibration of the analysers was performed according to manufacturer instructions.

**Fig. 1:**
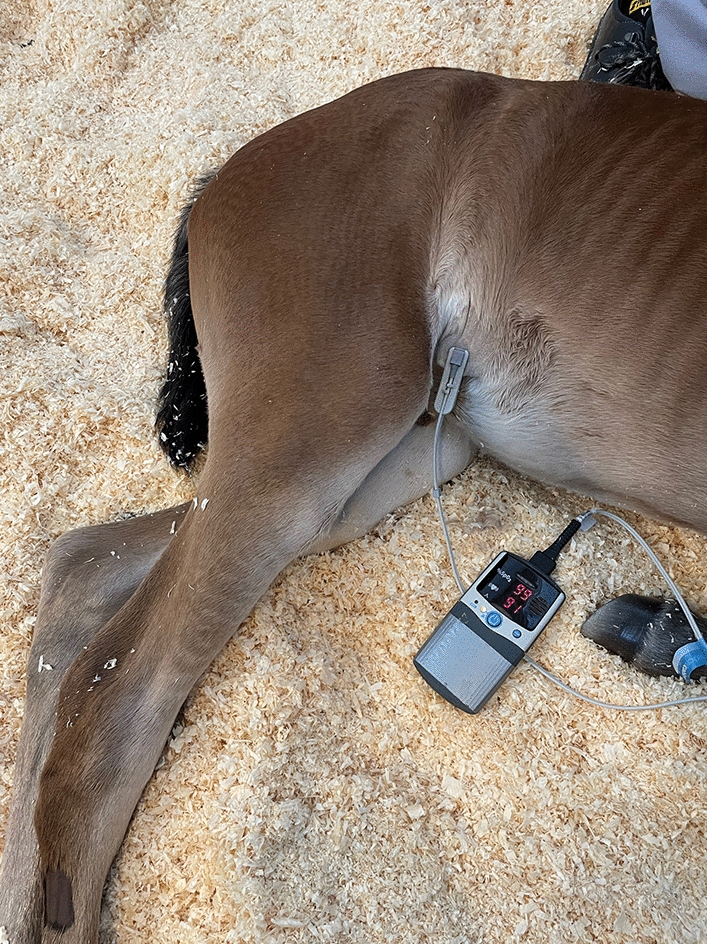
Pulse oximetry measurement from a foal’s skin fold. The pulse oximeter (Nonin PalmSAT 2500a VET, Jørgen Kruuse A/S, Langeskov, Denmark) transmission sensor is placed on a skin fold in caudal abdominal region of a recumbent, conscious foal

**Fig. 2:**
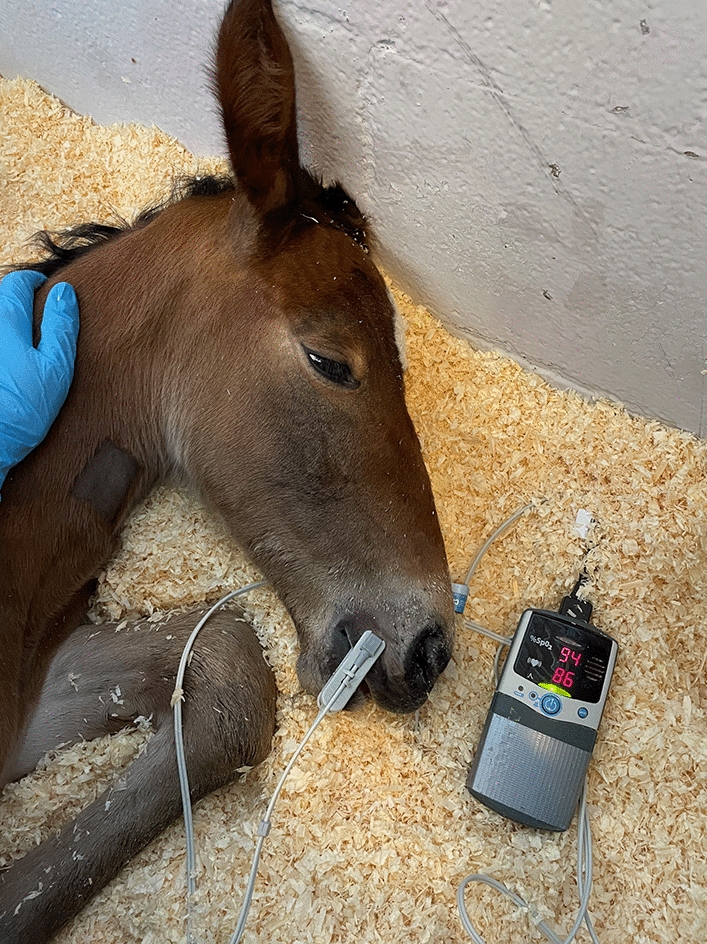
Pulse oximetry measurement from a foal’s upper lip. The pulse oximeter (Nonin PalmSAT 2500a VET, Jørgen Kruuse A/S, Langeskov, Denmark) transmission sensor is placed on upper lip of a recumbent, conscious foal

Immediately after arterial blood sample collection, while the foal was still in lateral recumbency, hair was clipped from a 5 × 5 cm area on the left or right caudal abdomen depending on the lying position (if it was not clipped already), and a transmission-type veterinary lingual pulse oximeter sensor was attached to an inguinal skin fold in a standardised manner after wetting the skin with water or skin disinfectant as needed (Nonin PalmSAT 2500a VET, Jørgen Kruuse A/S, Langeskov, Denmark; Fig. 1). This process took approximately 30 s to 2 min in total. Once a strong signal was detected and the pulse oximeter readings were consistent and stable with a green pulse indicator signal and without malfunction warnings, three consecutive SpO_2_ readings were recorded with a repeated sensor placement each time. Thereafter, for a subset of foals (14/19 for control foals and 2/9 for pneumonia foals), the same transmission sensor was placed on the foal’s upper lateral lip (Fig. 2) in a similar standardised manner, and three consecutive recordings were obtained. Pulse rate indicated by the pulse oximeter was ensured by thoracic auscultation to be equal to the auscultated heart rate. The whole sampling process was performed within approximately 5 min.

For pneumonia foals, one or two measuring sessions were performed daily during hospitalisation. During each session, arterial blood samples and pulse oximetry measurements were obtained. Control foals were similarly sampled once daily during their hospitalisation. The duration of repeated sampling was dependent on the length of hospitalisation which varied among foals. The foals examined at home stable were sampled only once. Moreover, for pneumonia foals, additional pulse oximetry measurements without arterial blood sampling in the same measuring session were performed in above-described manner up to four times per day depending on the foal’s clinical condition and their concurrent participation in another unrelated clinical study. For pneumonia foals requiring oxygen therapy, oxygen was turned off 15 min before the sample collection. Otherwise, the foals with pneumonia and control foals with a concurrent disease unrelated to the cardiovascular or respiratory system were allowed to receive all necessary treatments needed during their hospitalisation. Pulse oximeter measurements were performed by five different measurers trained to using the equipment (two veterinarians, three veterinary physiotherapists), and the measurers were not aware of the arterial blood sample results prior to performing the pulse oximetry.

### Statistical analysis

Statistical analyses were performed using statistical software SAS, version 9.4 (SAS Institute Inc., Cary, NC, USA). All analyses were conducted for the whole data and separately for pneumonia foals and controls. The number of measurements rather than foals was considered as the sample. Differences were considered significant at *P* < 0.05.

Computational arterial blood oxygen saturation (SaO_2_calc) was calculated based on the measurement of partial pressure of oxygen (PaO_2_) in arterial blood gas analysis using formulae described earlier [19]. The corrected PaO_2_ values, which also consider the foal’s body temperature, arterial blood pH, and partial pressure of carbon dioxide in arterial blood (PaCO_2_) were used in the calculation of SaO_2_calc. The SaO_2_calc and mean SpO_2_ were compared in terms of linear regression analysis, with SpO_2_ as the response and SaO_2_calc as the explanatory factor. Fit plots were generated to illustrate the association and model fit. In addition, Bland–Altman plots were generated to evaluate the agreement between SaO_2_calc and SpO_2_ [20]. Mean difference together with 95% limits of agreement were included in these plots. The comparison between SaO_2_calc and SpO_2_ was repeated separately for lip and skin fold measurements, and for pneumonia and control foals. Moreover, the effect of coat colour on SaO_2_calc and SpO_2_ difference was investigated with Kruskal–Wallis test.

The differences between the two measuring locations in the SpO_2_ measurements (not considering the foal effect) were evaluated using two approaches: (1) including all SpO_2_ measurements (all repeats), and (2) calculating the mean of the three SpO_2_ measurements within measuring session first and then comparing these means between the locations. SpO_2_ measurement results between the two locations (lip, skin fold) were formally compared with a Mann–Whitney U-test.

The difference between the two measuring locations (lip, skin fold) within the foal was evaluated using a subset of the data where measurements from both locations had been obtained during the same measuring session successively. The differences between lip and skin fold were calculated from this paired data and formally analysed by Wilcoxon Signed Rank test.

To evaluate the repeatability of the pulse oximeter SpO_2_ measurements, the intrarater reliability (random variation within measurer) was assessed separately for the two measuring locations (lip, skin fold). Intrarater reliability was evaluated based on the three repeated measurements at each measuring session within the measuring location. Intraclass correlation coefficients (ICCs) with 95% confidence intervals (CIs) were calculated to assess the internal consistency between the repeats within the measurer. In addition, the intrarater reliability evaluations were repeated using only 2 of the 3 repeated measurements (the middle-measured value and another measured value closest to the middle value).

## Results

The mean age (± SD, range) of the foals was 5 days (± 3.7, 1.0–16.0 days), and 19/32 foals were under 5 days of age on admission. Breeds included were Standardbred (n = 9), Finnhorse (n = 9), Warmblood (n = 12), one mixed-breed draft horse, and one pony. On average, the foals weighed 56.2 kg (± 14.5, 20.0–90.0 kg), and the coat colours varied, including bay (n = 14), dark and light chestnut (n = 8 for both), and black (n = 2). Characteristics of the enrolled foals per group are presented in Table 1.

**Table 1:** Characteristics of the foals included in the study

Variable		Pneumonia	Control
Age (days)	n	13	19
	Mean (± SD)	5.8 (± 4.7)	4.5 (± 2.9)
	Min–max	1.5–16.0	1.0–10.0
Weight (kg)	n	12	10
	Mean (± SD)	53.7 (13.7)	59.1 (15.7)
	Min–max	20.0–70.0	35.0–90.0
Sex (n, %)	Filly	3 (23.1)	11 (57.9)
	Colt	10 (76.9)	8 (42.1)
Breed (n, %)	Finnhorse	5 (38.5)	4 (21.1)
	Mixed-breed draft horse		1 (5.3)
	Pony	1 (7.6)	
	Standardbred	4 (30.8)	5 (26.3)
	Warmblood	3 (23.1)	9 (47.3)
Colour (n, %)	Bay	6 (46.1)	8 (42.1)
	Black		2 (10.5)
	Dark chestnut	3 (23.1)	5 (26.3)
	Light chestnut	4 (30.8)	4 (21.1)

Description of the two groups of foals in the study (foals with pneumonia and control foals with normal respiratory and cardiovascular status)

*SD* standard deviation, *n* number of foals

Altogether 93 arterial blood samples were taken that were paired with pulse oximetry measurements from either lip, skin fold or both, measured in the same session, resulting in 93 pairs in total. In addition, for a subgroup of foals (14/19 for control foals and 2/9 for pneumonia foals), consecutive SpO_2_ measurements (n = 58) from two anatomical sites (lip, skin fold) within the same measuring session were assessed. For the assessment of repeatability, a further 437 pulse oximetry measurements were evaluated without a same measuring session arterial blood sample. In total, independent pulse oximetry measurement was performed 196 times from the foal’s lip and 338 times from an abdominal skin fold. For pneumonia foals, PaO_2_ varied from 35.7 mmHg to 86.0 mmHg, with a mean of 60.6 (± 10.8) mmHg, and for control foals PaO_2_ varied from 59.1 mmHg to 94.5 mmHg, with a mean of 75.8 (± 10.0) mmHg. PaCO_2_ varied from 30.9 mmHg to 56.6 mmHg, with a mean of 44.9 (± 5.6) mmHg, in pneumonia foals and from 36.5 mmHg to 46.4 mmHg, with a mean of 40.7 (± 3.3) mmHg, in control foals.

In general, pulse oximetry was well tolerated in conscious foals. For some foals, pulse oximetry was challenging due to the foal’s demeanour or movement attempts, especially when measured from the lip, with a longer wait to obtain a reliable result with a green signal indicator and stable pulse reading in the device. On some occasions, replacement of the sensor or moistening of the lip mucous membrane or skin was required to achieve sufficient contact. Skin fold measurements were generally well tolerated in recumbent foals, and stable readings were easier to obtain. However, there was variability among foals regarding the two measuring locations; for some foals, the lip was superior to the skin fold regarding ease of obtaining reliable readings, and vice versa.

### Comparison of pulse oximetry-measured saturation and calculated saturation

**Fig. 3:**
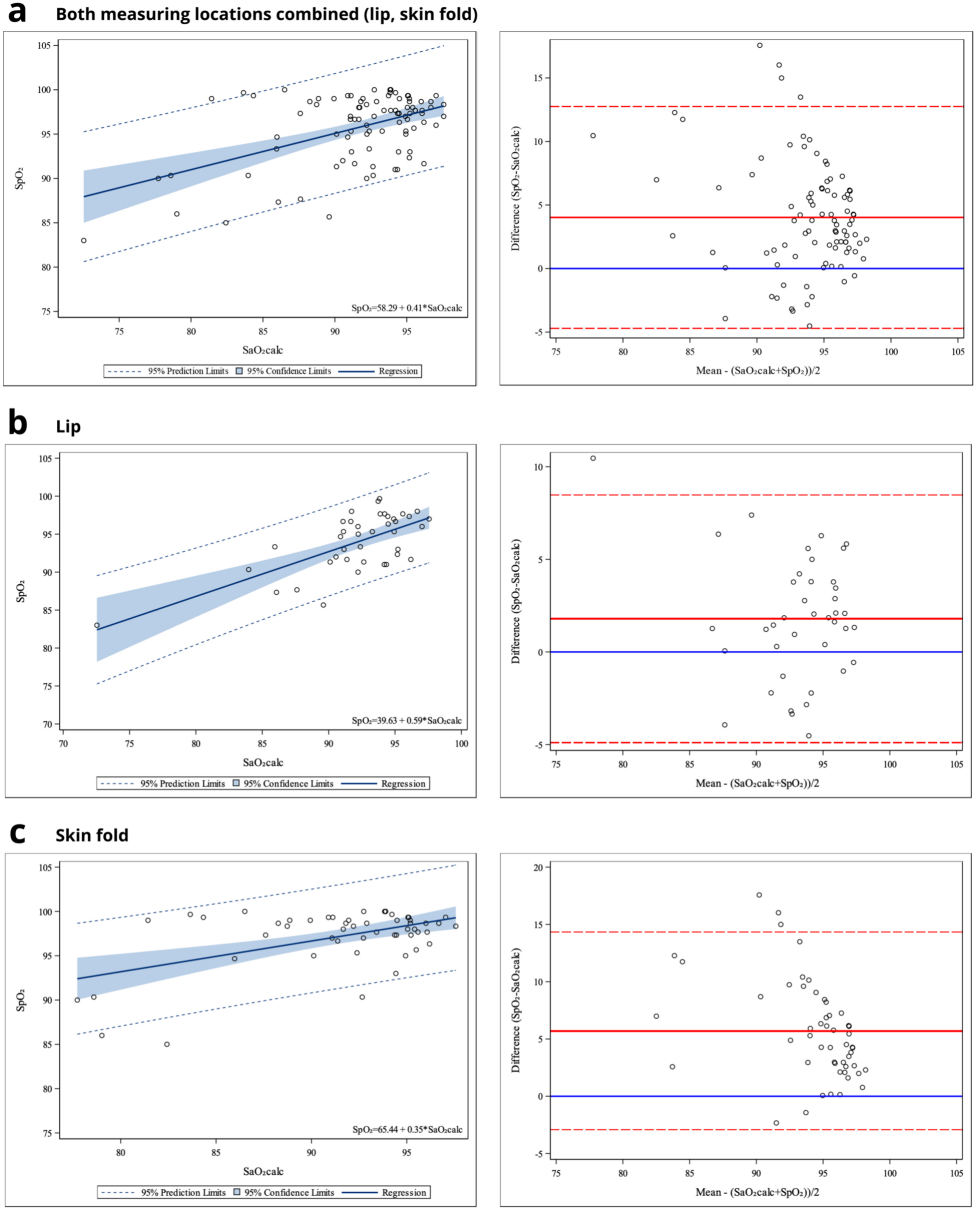
Linear regression fit plots and Bland–Altman plots for measured and calculated blood oxygen saturation. For assessing the method association and agreement, linear regression fit plots and Bland–Altman scatter plots comparing the oxygen saturation of arterial blood detected by pulse oximeter (SpO_2_) and computational oxygen saturation of arterial blood (SaO_2_calc) based on arterial blood gas analysis are presented for 93 paired measurements. Data for all foals (n = 32) and both pulse oximetry measuring locations (lip, skin fold) combined are presented (**a**), as are data for lip (**b**) and skin fold (**c**) measuring locations separately. In scatter plots with trend lines for linear regression, the shadowed area represents the 95% confidence limits and dashed lines indicate the 95% prediction limits. The estimated regression line formula is presented. In Bland–Altman scatter plots, the horizontal solid red line represents the mean difference between the two measures. The two dashed red lines represent the 95% limits of agreement (mean difference ± 1.96 standard deviation of the difference). The solid blue line is the line of equality, which indicates the same value between the two measures

Based on regression analysis on 93 paired arterial blood samples and pulse oximetry measurements, SaO_2_calc and SpO_2_ were clearly associated with each other (Fig. 3 and Additional file 1). However, when using an average of all three SpO_2_ repeats of all foals, the SpO_2_ by the pulse oximeter was 4.0 (± 4.4) percentage units higher on average than SaO_2_calc. When comparing the SaO_2_calc and SpO_2_ within the two locations (lip, skin fold), SaO_2_calc and SpO_2_ matched better with each other when the measurement was obtained from the lip; the difference was 1.8 (± 3.3) percentage units for the lip and 5.7 (± 4.3) percentage units for the skin fold (SaO_2_calc < SpO_2_, Fig. 3). Thus, especially the skin fold measurements overestimated the saturation relative to the calculated value, indicating systematic bias, which was clearly less pronounced in lip measurements.

When analysing pneumonia and control foals separately, the SpO_2_ by the pulse oximeter was 5.3 (± 5.1) percentage units higher on average than SaO_2_calc for pneumonia foals, and 2.4 (± 2.5) percentage units for control foals (Additional file 1). However, the number of skin fold measurements was proportionally higher than the number of lip measurements in pneumonia foals compared with controls (n = 31 for skin fold and n = 20 for lip in pneumonia foals; n = 22 for skin fold and n = 20 for lip in control foals). Nine paired values (out of 93 pairs used in the analysis) were found to differ even more than ten percentage units; range of variation was from 10.1 percentage units to up to 17.6 percentage units (SaO_2_calc < SpO_2_). These outliers were found in four foals: three measurements in foal number 1, three in foal number 4, one in foal number 5, and two in foal number 9. Foal 1 was bay colour, foal 4 was light chestnut, foal 5 was bay, and foal 9 was dark chestnut. All of these foals belonged to the pneumonia group. There was no clear explanation for the marked difference between SpO_2_ and SaO_2_calc in these four foals.

### Effect of coat colour on pulse oximetry-measured saturation versus calculated saturation

No significant differences were found between the colours regarding SpO_2_ and SaO_2_calc in lip measurements. For skin fold measurements, the only colours with a significant difference (P = 0.0081) between them in SpO_2_ and SaO_2_calc results were bay and dark chestnut, with a mean difference percentage of 6.5 (± 4.2) and 1.7 (± 2.6), respectively.

### Comparison of lip and skin fold saturation measurement results

When comparing the mean SpO_2_ between lip and skin fold measurements (all measurements in all foals included, without considering foal effect), the measurements from the skin fold were on average 2.9 percentage units higher than the measurements from the lip (Table 2). Similar results were achieved in analysing the difference between locations within a foal when measurements obtained in the same measuring session only were considered; the average difference between lip and skin fold SpO_2_ was 2.4 percentage units (lip < skin fold, Table 2).

**Table 2:** Comparison of arterial blood oxygen saturation (SpO_2_) measured from two locations (lip and skin fold)

Group	Variable	Statistics	Lip	Skin fold	Overall	P-value
All foals	SpO_2_ all repeats	n	196	338	534	
		Mean (± SD)	94.2 (± 3.7)	97.1 (± 3.4)	96.0 (± 3.8)	< 0.0001*
		Min–max	82.0–100.0	80.0–100.0	80.0–100.0	
	Mean SpO_2_	n	66	122	188	
		Mean (± SD)	94.2 (± 3.4)	97.0 (± 3.4)	96.0 (± 3.7)	< 0.0001*
		Min–max	83.0–100.0	82.0–100.0	82.0–100.0	
	Diff skin–lip within foal	n			58	
		Mean (± SD)			2.4 (± 2.4)	< 0.0001**
		Min–max			–1.0–8.0	
Pneumonia	SpO_2_ all repeats	n	136	274	410	
		Mean (± SD)	93.4 (± 3.9)	96.9 (± 3.7)	95.8 (± 4.1)	< 0.0001*
		Min–max	82.0–100.0	80.0–100.0	80.0–100.0	
	Mean SpO_2_	n	46	100	146	
		Mean (± SD)	93.4 (± 3.6)	96.8 (± 3.6)	95.8 (± 3.9)	< 0.0001*
		Min–max	83.0–100.0	82.0–100.0	82.0–100.0	
Controls	SpO_2_ all repeats	n	60	64	124	
		Mean (± SD)	96.0 (± 2.6)	98.0 (± 1.6)	97.0 (± 2.4)	< 0.0001*
		Min–max	91.0–100.0	95.0–100.0	91.0–100.0	
	Mean SpO_2_	n	20	22	42	
		Mean (± SD)	96.0 (± 2.4)	98.1 (± 1.4)	97.1 (± 2.2)	0.0028*
		Min–max	91.7–99.7	95.0–100.0	91.7–100.0	

^*^by Mann–Whitney U-test, ** by Wilcoxon Signed Rank test

Results for all foals (n = 32) and separately for pneumonia foals (n = 13) and controls (n = 19) are indicated. Results are presented considering all SpO_2_ measurements (SpO_2_ all repeats) and the mean of the three SpO_2_ measurements within the measuring session (Mean SpO_2_). Difference in SpO_2_ between skin fold and lip measurements within foal (Diff skin–lip within foal) obtained in the same measuring session is presented. Means, standard deviations (SD) and minimum and maximum values are displayed as percentage units. P-values are indicated for the difference between lip and skin fold measurement values. *n* number of measurements

### Repeatability of pulse oximetry measurements

The internal consistency of the three repeats of SpO_2_ measurement was moderate to good [21] for both skin fold and lip measurements considering all foals. The overall ICC and 95% CI was 0.695 (0.633–0.747) for skin fold and 0.798 (0.741–0.843) for lip. However, ICC was found to vary significantly between different measurers (0.377–0.859), and also the number of measuring sessions (each including three measurements) was variable between measurers for both measuring locations (2–33 for lip and 7–43 for skin fold; Additional file 2). When using only two of the three repeated SpO_2_ measurement results (the middle value and the one closest to the middle value), ICC markedly improved, being 0.962 (0.946–0.974) for skin fold and 0.978 (0.965–0.987) for lip, indicating minimal variability and excellent reliability [21]. When the three repeated measurements and their order were assessed further considering only the occasions where at least a 3 percentage unit difference from the middle value of the measurements was detected (n = 50), we found that for 44% of measurement occasions the most deviating measurement was the first measurement, 24% the second measurement, and 32% the third measurement. The internal consistency results per measurer and per group for both triplicate and duplicate measurements are presented in Additional files 2 and 3, respectively.

## Discussion

This study evaluated the overall usability and, more precisely, the validity and reliability of pulse oximetry at two measuring sites (lip and skin fold) in conscious foals with and without respiratory compromise in a clinical setup. Pulse oximetry has been considered challenging in awake foals [1], but this is the first study to evaluate transmission pulse oximetry at different measuring sites in conscious foals. We found that pulse oximetry measurement from the lip seems to be valid in assessing the oxygenation of awake foals with low bias level compared with calculated saturation values based on arterial blood samples. However, pulse oximetry may overestimate the saturation if the measurement is performed from the caudal abdominal skin fold. Measurements obtained from the lip relative to the skin fold were more accurate and reliable, although skin fold measurement was technically easier to perform in many foals. The benefits of pulse oximetry compared with invasive arterial blood sampling include the ability to obtain a rapid, real-time, and pain-free assessment of the foal’s oxygenation. Although pulse oximetry alone is not sufficient to determine the oxygenation of the foal, with arterial blood sampling being the gold standard, pulse oximetry can help in on-site determination of the need for supplemental oxygen therapy, and it may aid in monitoring the immediate effect of therapeutic procedures.

We found that lip SpO_2_ measurements overestimated the saturation by less than 2 percentage units on average, which is within the pulse oximeter manufacturer’s claim of accuracy (± 2% over the range of SpO_2_ 70–100%) and within the generally accepted typical accuracy of ± 2–4% for pulse oximeters [6]. However, skin fold measurements overestimated saturation even more than 5 percentage units. A human study used a portable microspectrometer to evaluate the accuracy of different pulse oximeters and found that a significant proportion of pulse oximeters were inaccurate with an error of over 4 percentage units in a saturation range of 70–100% [22], which can indicate clinically significant validity issues. An anaesthesia study regarding pulse oximeters in several animal species showed that accuracy varied between 2 and 5% in horses [23], in agreement with our results.

The clinical significance of the difference between measured SpO_2_ and calculated SaO_2_ observed in our study can be argued. Considering the oxygen-haemoglobin dissociation curve, a saturation bias of approximately 2 percentage units, as found in the lip measurements in this study, might not yet be clinically relevant. However, a bias of over 5 percentage units can be alarming and of clinical significance, and due to the sigmoid shape of the oxygen-haemoglobin dissociation curve, the potential effect on tissue oxygenation of an erroneous reading is even greater at lower saturation levels. Studies in hypoxaemic children have similarly found that pulse oximetry typically overestimates SaO_2_ [24–26], but bias may vary according to the level of hypoxaemia and the measuring site [25, 27]. The effect of the degree of hypoxaemia on the bias level of SpO_2_–SaO_2_calc was not investigated in this study, as most saturation values were in the upper end of the oxygen-haemoglobin dissociation curve. Because the study was performed in awake foals only, this was expected. In conclusion, pulse oximetry from the lip seems to provide a useful estimation of the oxygenation status in conscious foals in clinical practice, taking into consideration the potential small saturation overestimation, but skin fold measurement cannot be deemed clinically useful based on our results.

In previous studies in dogs and foals, pulse oximeter probe placement site has been found to contribute to the discrepancy between measured SpO_2_ and SaO_2_ [16, 17, 28]. Pulse oximetry from the lip or tongue has previously been reported to be the most accurate in assessing SaO_2_ in anaesthetised foals [17] and dogs [28, 29]. Thus, the lip was selected as the other measuring site in this study since the tongue was considered ineligible in conscious foals in practice. However, pulse oximetry from the lip overestimated SaO_2_ over all ranges of saturation in anaesthetised foals [16], which could be observed also in our results in conscious foals without a desaturation protocol, but the level of overestimation in lip measurements in our study was low. In one study evaluating the accuracy of reflectance pulse oximetry SpO_2_ measurements at the base of the tail relative to calculated SaO_2_ in conscious foals, a mean difference of 2.5 ± 3.5% was found between SpO_2_ and SaO_2_ [18], which is approximately the difference level found in our study when the measurement was performed from the lip.

Skin fold was chosen as the second measuring site due to the easy placement of the skin in the caudal abdominal area between the pulse oximeter’s sensor clamp and the assumed easy implementation of the method in this area in conscious foals. Pulse oximetry from a skin fold has not been investigated in foals before, but it was inferior regarding accuracy relative to lip measurements in dogs in a study evaluating multiple measuring sites and two probes [29], similarly to the results obtained here. Furthermore, in that study, the pulse oximeter often failed to provide readings from a dog’s flank skin fold. It was speculated that the tissue between the light-emitting diode and the photosensor of the probe could have been too thick or the perfusion of the area poorer than in other measuring sites [29]. These reasons are possible also in foals, although in our study obtaining successful readings was generally not an issue but rather the overestimation of the saturation by pulse oximetry from the skin fold compared with calculated saturation and with lip measurements. This was more pronounced in pneumonia foals relative to control foals, indicating that the potentially decreased perfusion of the peripheral tissue in foals suffering from pneumonia might have affected the accuracy of skin fold measurements. This might have also affected one of the outcomes of our study, where a larger difference between SpO_2_ and calculated SaO_2_ without considering measuring location was found in pneumonia foals than in control foals.

Pulse oximeter performance influences clinical decisions in patient care; pulse oximetry is not only used in assessment of oxygenation during clinical examination of the patient to detect need for oxygen supplementation, but it is also applied to determine trends in a patient's blood oxygen saturation levels during monitoring to guide treatment decisions. Thus, pulse oximetry can also be useful in warning clinicians of declining saturation levels. The level of pulse oximeter accuracy found in this study is probably sufficient to detect a significant deterioration in the oxygenation of the conscious foal, especially when measured from the lip, which is clinically more important than the absolute precision of the method. In clinical practice, diagnosis and treatment decisions are, or at least should be, determined by monitoring the pulse oximeter readings over time, rather than following any absolute thresholds. However, saturation level changes and how well pulse oximetry as a method would perform in detecting these changes in conscious foals were not assessed here and would be a valuable but challenging topic for future research.

The consistency (reliability) of the repeated pulse oximeter measurements was moderate to good [21] if triplicate measurements were considered and improved significantly after discarding the most deviating measurement. When the deviating measurement was omitted, the remaining measurements matched well, indicating that the reliability issue seemed to involve only one of the three repeated measurements. However, the proportion of deviating measurements with a 3 percentage unit difference or more from the middle value of the measurements was generally low. The largest proportion of the most deviating measurements was obtained at the first measurement, with the second measurement being most accurate. This indicates that there may have been a technical or operator-related reason involved in the discrepancy of the first measurement rather than any foal-related reason. Thus, although pulse oximetry as a method may be rather reliable, there is a risk of human error in the measurement process. A potential reason for this may be poor contact on the first measurement, not waiting sufficiently long for the reading to stabilise or placing the probe on a pigmented spot of the lip the first time, among others. This should be considered when performing pulse oximetry in conscious foals. It might be advisable in clinical practice to obtain serial measurements to determine whether the results are consistent and the pulse oximeter gives a reliable signal. Moreover, although in this study the potential pigment on the inner surface of the lip was not inspected or recorded, in clinical practice it may be wise to avoid pigmented areas, if possible. Pigment has been found to interfere with the accuracy of pulse oximetry readings in humans, resulting in overestimation of saturation [30], and dark pigment is assumed to interfere with pulse oximetry measurements also in foals [31]. In this study, foals with different coat colours were used, but no clinically relevant discrepancies between measured SpO_2_ versus calculated SaO_2_ were found between different colours within the two pulse oximetry measuring locations. Thus, coat colour does not seem to affect pulse oximetry results in foals, however, the effect of black coat colour could not be evaluated in this study due to their low number.

There is no true gold standard for arterial blood oxygen saturation assessment in horses, i.e. it cannot be measured directly from the animal’s blood real-time. The SaO_2_ result provided by the blood gas analyser, which was used in this study, is based on algorithms in humans, as is the case for many other analysers used in veterinary clinical practice and research. Therefore, for comparison of the measured SpO_2_, the calculated saturation based on adult equine data was used as a reference method, and it was computed according to a previous publication [19]. It has been found that calculated SaO_2_ is as accurate as SpO_2_ in assessing the SaO_2_ defined by co-oximetry in anaesthetised foals, but particularly at low saturation levels the adult equine formula might be less accurate than a human data-based formula as a reference method [17]. However, most of the saturations detected in our study were not low since conscious foals were used. The formulae used in the calculation of SaO_2_ take into account the PaO_2_, PaCO_2_ and pH in arterial blood gas analysis and the horse’s body temperature [19]. Yet, the formula provides only a calculated estimate, and the real-life situation might be somewhat different, which might have influenced the results obtained in this study. The same formula has been used in previous publications related to pulse oximetry in horses [17, 18, 32] and found to be accurate with PaO_2_ levels over 80 mmHg [33]. Thus, it was deemed acceptable to use also in this study for comparing methods despite the potential sources of inaccuracy. Furthermore, there is no commercially available pulse oximeter calibrated for equine blood. Even though the pulse oximeter used in this study (Nonin PalmSAT 2500a VET) is designed for veterinary use, according to the manufacturer the accuracy is specified based on healthy human adult haemoglobin. However, haemoglobin of animals has been found to have similar optical characteristics to humans [34]. Contrary to many other species, the haemoglobin of the newborn foal and the adult horse is reported to be structurally similar [35] but with higher affinity to oxygen than maternal blood in foals under 5 days of age due to lower red blood cell 2,3-diphospho-glycerate levels [36]. Thus, the blood of foals included in this study which were under 5 days of age on admission (n = 19/32) may have influenced the accuracy results due to physiological reasons and due to the lack of an equine-calibrated pulse oximeter. The effect of haemoglobin concentration of the foals on pulse oximetry was not explored in this study.

There are some further limitations in this study. Due to technical and clinical patient care-related reasons, the arterial blood gas sampling and pulse oximetry were not performed exactly at the same time but sequentially within minutes from each other with the same restraint of the foal. For a few foals that resisted the sampling procedures, the successful sampling took longer than for other foals. This could have affected the results. Moreover, the order of skin fold and lip pulse oximetry measurements was not random, as skin fold measurements were always performed first and lip measurements second to avoid provoking the foal due to expected stronger reaction to lip measurements. This could be a source of methodological bias and influence the results of this study, since prolonged lateral recumbency might decrease the PaO_2_ and oxygen saturation values but not linearly due to the shape of the oxygen-haemoglobin dissociation curve. Thus, the effect on the calculated saturation (based on PaO_2_, among other factors) and the comparison between lip and skin fold pulse oximetry is possible and could not be controlled. The assessment of validity and reliability of pulse oximetry was not possible over a large scale of blood oxygen saturation levels, as it would be in anaesthesia studies using desaturation protocols; the majority of the measured values were in the top end of the saturation scale in conscious foals, and there was a relatively small number of hypoxaemic foals included. Pulse oximetry was performed by five different measurers, two veterinarians and three veterinary physiotherapists (due to another concurrent trial), trained to use the equipment. However, some of the measurers might not have been aware of the clinical significance of the acquired values at the time of the pulse oximetry measurement. Moreover, as the number of triplicate measurements was very limited for some measurers from one of the two measuring locations (2 at minimum), even a single clearly deviating measurement could have had a considerable impact on the intrarater reliability assessment results. Thus, the results between measurers were not fully comparable. Furthermore, interrater reliability of the pulse oximetry measurements could not be assessed since only one person at a time performed the measurement. Defining interrater reliability would add to the value of reliability assessment in future studies. We did not have access to an equine-specific co-oximeter for assessing arterial blood oxygen saturation, and the limited availability of co-oximetry for equine studies remains a challenge. In future studies, pulse oximetry-measured SpO_2_ should be compared also with saturation defined by co-oximeter in conscious foals. Although a veterinary pulse oximeter was utilised in this study, some level of inaccuracy must be expected, especially when assessing ailing animals due to calibration of the device being performed based on data of healthy humans. Furthermore, the pulse oximetry was performed in conventional lighting conditions in a hospital or at a home stable, and the influence of ambient light on the SpO_2_ measurements could not be excluded in this study. The use of a portable blood gas analyser for the four foals sampled at the home stable remains a potential limitation of this study despite the uniformity of the blood gas analysis results of both analysers was tested prior to sampling to provide comparable results (no data available).

## Conclusions

Pulse oximetry with the sensor attached to the lip is a clinically applicable and valid method in conscious foals for arterial blood oxygen saturation determination, but pulse oximetry from skin fold is unreliable. Regarding repeatability, several measurements should be obtained and outliers discarded to obtain a reliable result.

## Supplementary Information

Additional file 1. Linear regression fit plots and Bland-Altman plots for measured and calculated blood oxygen saturation in two groups of foals (pneumonia and controls). For assessing the method association and agreement, linear regression fit plots and Bland-Altman scatter plots comparing the oxygen saturation of arterial blood detected by pulse oximeter (SpO_2_) and computational oxygen saturation of arterial blood (SaO_2_calc) based on arterial blood gas analysis are presented for 93 paired measurements. Data for foals with pneumonia (a; n=13) and control foals with normal respiratory and cardiovascular function (b; n=19) are presented, and both pulse oximetry measuring locations (lip, skin fold) are presented together. In scatter plots with trend lines for linear regression, the shadowed area represents the 95% confidence limits and dashed lines indicate the 95% prediction limits. The estimated regression line formula is presented. In Bland-Altman scatter plots, the horizontal solid red line represents the mean difference between the two measures. The two dashed red lines represent the 95% limits of agreement (mean difference ± 1.96 standard deviation of the difference). The solid blue line is the line of equality, which indicates the same value between the two measures.

Additional file 2. Internal consistency of pulse oximetry triplicate measurements. Internal consistency results per measurer and per group for triplicate pulse oximetry measurements are presented. Results for all foals (n=32) and separately for pneumonia foals (n=13) and controls with normal respiratory and cardiovascular function (n=19) are indicated. Five different measurers (Meas 1 to Meas 5) performed the pulse oximetry, and the number of measuring sessions (N of meas) is indicated. *ICC* intraclass correlation coefficient, *CI* confidence interval.

Additional file 3. Internal consistency of pulse oximetry duplicate measurements. Internal consistency results of pulse oximetry duplicate measurements, including two of the three repeated SpO_2_ measurement results (the middle and the one closest to the middle), are presented. Results for all foals (n=32) and separately for pneumonia foals (n=13) and controls with normal respiratory and cardiovascular function (n=19) are indicated. Five different measurers (Meas 1 to Meas 5) performed the pulse oximetry, and the number of duplicate measuring sessions (N of meas) is indicated. *ICC* intraclass correlation coefficient, *CI* confidence interval.

## Data Availability

The data supporting the findings of this study are not openly available due to reasons of confidentiality (patient data) but are available from the corresponding author upon reasonable request.

## Abbreviations

CI: Confidence interval
ICC: Intraclass correlation coefficient
PaCO_2_: Partial pressure of carbon dioxide in arterial blood
PaO_2_: Partial pressure of oxygen in arterial blood
SaO_2_: Oxygen saturation of arterial blood
SaO_2_calc: Calculated oxygen saturation of arterial blood
SD: Standard deviation
SpO_2_: Oxygen saturation of arterial blood detected by pulse oximeter

## Declarations

### Prior publication

Data have not been published previously.
