# Supplementary material for: Pulse oximetry at two sensor placement sites in conscious foals
Source: Acta Vet Scand. 2025 Jan 23;67:6. doi: 10.1186/s13028-025-00794-w (PMC11761757; doi:10.1186/s13028-025-00794-w)
Supplement: Supplementary file 1 — Additional file 1. Linear regression fit plots and Bland-Altman plots for measured and calculated blood oxygen saturation in two groups of foals (pneumonia and controls). For assessing the method association and agreement, linear regression fit plots and Bland-Altman scatter plots comparing the oxygen saturation of arterial blood detected by pulse oximeter (SpO2) and computational oxygen saturation of arterial blood (SaO2calc) based on arterial blood gas analysis are presented for 93 paired measurements. Data for foals with pneumonia (a; n=13) and control foals with normal respiratory and cardiovascular function (b; n=19) are presented, and both pulse oximetry measuring locations (lip, skin fold) are presented together. In scatter plots with trend lines for linear regression, the shadowed area represents the 95% confidence limits and dashed lines indicate the 95% prediction limits. The estimated regression line formula is presented. In Bland-Altman scatter plots, the horizontal solid red line represents the mean difference between the two measures. The two dashed red lines represent the 95% limits of agreement (mean difference ± 1.96 standard deviation of the difference). The solid blue line is the line of equality, which indicates the same value between the two measures. [file 13028_2025_794_MOESM1_ESM.pdf]

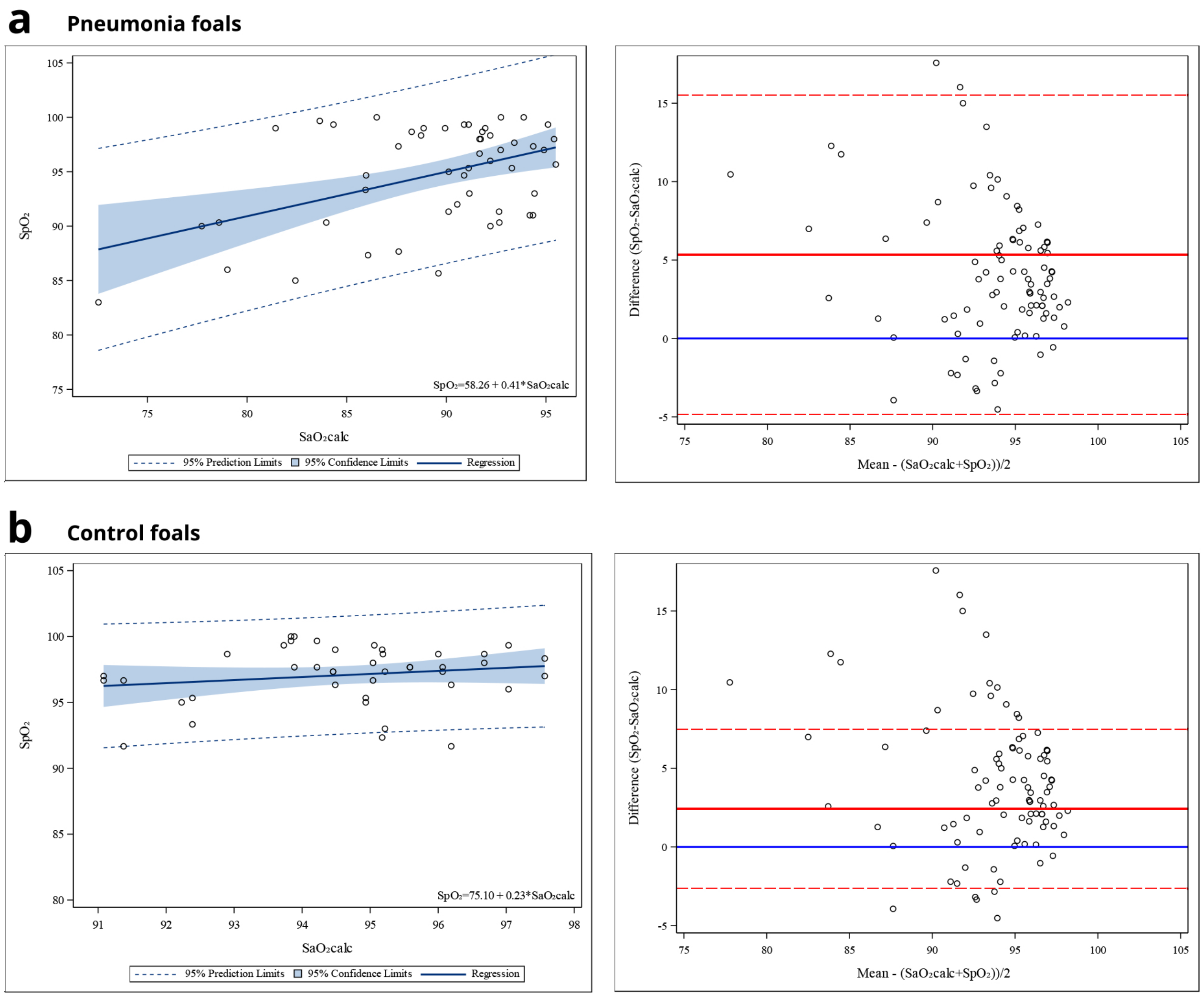

Additional file 1. Linear regression fit plots and Bland-Altman plots for measured and calculated blood oxygen saturation in two groups of foals (pneumonia and controls).

For assessing the method association and agreement, linear regression fit plots and Bland-Altman scatter plots comparing the oxygen saturation of arterial blood detected by pulse oximeter (SpO_2) and computational oxygen saturation of arterial blood (SaO_2calc) based on arterial blood gas analysis are presented for 93 paired measurements. Data for foals with pneumonia (a; n=13) and control foals with normal respiratory and cardiovascular function (b; n=19) are presented, and both pulse oximetry measuring locations (lip, skin fold) are presented together. In scatter plots with trend lines for linear regression, the shadowed area represents the 95% confidence limits and dashed lines indicate the 95% prediction limits. The estimated regression line formula is presented. In Bland-Altman scatter plots, the horizontal solid red line represents the mean difference between the two measures. The two dashed red lines represent the 95% limits of agreement (mean difference ± 1.96 standard deviation of the difference). The solid blue line is the line of equality, which indicates the same value between the two measures.
