# Supplementary material for: Pulse oximetry at two sensor placement sites in conscious foals
Source: Acta Vet Scand. 2025 Jan 23;67:6. doi: 10.1186/s13028-025-00794-w (PMC11761757; doi:10.1186/s13028-025-00794-w)
Supplement: Supplementary file 2 — Additional file 2. Internal consistency of pulse oximetry triplicate measurements. Internal consistency results per measurer and per group for triplicate pulse oximetry measurements are presented. Results for all foals (n=32) and separately for pneumonia foals (n=13) and controls with normal respiratory and cardiovascular function (n=19) are indicated. Five different measurers (Meas 1 to Meas 5) performed the pulse oximetry, and the number of measuring sessions (N of meas) is indicated. ICC intraclass correlation coefficient, CI confidence interval. [file 13028_2025_794_MOESM2_ESM.pdf]

Additional file 2. Internal consistency of pulse oximetry triplicate measurements.
Internal consistency results per measurer and per group for triplicate pulse oximetry measurements are presented. Results for all foals (n=32) and separately for pneumonia foals (n=13) and controls with normal respiratory and cardiovascular function (n=19) are indicated. Five different measurers (Meas 1 to Meas 5) performed the pulse oximetry, and the number of measurement sessions (N of meas) is indicated. ICC=intraclass correlation coefficient, CI=confidence interval.

					95% CI	
**Group**	**Location**	**Measurer**	**N of meas**	**ICC**	**Lower**	**Upper**
**All foals**	Lip	Overall*	66	0.798	0.741	0.843
		Meas1	33	0.816	0.739	0.873
		Meas2	12	0.804	0.653	0.893
		Meas3	17	0.377	0.120	0.587
	Skin fold	Overall	106	0.695	0.633	0.747
		Meas1	43	0.463	0.317	0.588
		Meas2	13	0.588	0.344	0.758
		Meas3	7	0.859	0.694	0.938
		Meas4	8	0.491	0.133	0.736
		Meas5	35	0.749	0.651	0.822
**Pneumonia**	Lip	Overall*	46	0.793	0.722	0.847
		Meas1	25	0.816	0.724	0.879
		Meas3	17	0.377	0.120	0.587
	Skin fold	Overall	85	0.694	0.625	0.753
		Meas1	35	0.435	0.268	0.577
		Meas3	7	0.859	0.694	0.938
		Meas4	8	0.491	0.133	0.736
		Meas5	35	0.749	0.651	0.822
**Controls**	Lip	Overall	20	0.727	0.584	0.826
		Meas1	8	0.661	0.371	0.833
		Meas2	12	0.804	0.653	0.893
	Skin fold	Overall	21	0.646	0.478	0.768
		Meas1	8	0.723	0.470	0.866
		Meas2	13	0.588	0.344	0.758

** The data from measurers with ≤ 2 measurement sessions are not presented separately but are included in the "Overall" results.*
