# Supplementary material for: Pulse oximetry at two sensor placement sites in conscious foals
Source: Acta Vet Scand. 2025 Jan 23;67:6. doi: 10.1186/s13028-025-00794-w (PMC11761757; doi:10.1186/s13028-025-00794-w)
Supplement: Supplementary file 3 — Additional file 3. Internal consistency of pulse oximetry duplicate measurements. Internal consistency results of pulse oximetry duplicate measurements, including two of the three repeated SpO2 measurement results (the middle and the one closest to the middle), are presented. Results for all foals (n=32) and separately for pneumonia foals (n=13) and controls with normal respiratory and cardiovascular function (n=19) are indicated. Five different measurers (Meas 1 to Meas 5) performed the pulse oximetry, and the number of duplicate measuring sessions (N of meas) is indicated. ICC intraclass correlation coefficient, CI confidence interval. [file 13028_2025_794_MOESM3_ESM.pdf]

Additional file 3. Internal consistency of pulse oximetry duplicate measurements.
Internal consistency results of pulse oximetry duplicate measurements, including two of the three repeated SpO_2 measurement results (the middle and the one closest to the middle), are presented. Results for all foals (n=32) and separately for pneumonia foals (n=13) and controls with normal respiratory and cardiovascular function (n=19) are indicated. Five different measurers (Meas 1 to Meas 5) performed the pulse oximetry, and the number of duplicate measurement sessions (N of meas) is indicated. ICC=intraclass correlation coefficient, CI=confidence interval.

					95% CI	
**Group**	**Location**	**Measurer**	**N of meas**	**ICC**	**Lower**	**Upper**
**All foals**	Lip	Overall*	66	0.978	0.965	0.987
		Meas1	33	0.984	0.968	0.992
		Meas2	12	0.977	0.929	0.993
		Meas3	17	0.887	0.728	0.955
	Skin fold	Overall	106	0.962	0.946	0.974
		Meas1	43	0.953	0.917	0.974
		Meas2	13	0.937	0.820	0.979
		Meas3	7	1.000		
		Meas4	8	1.000		
		Meas5	35	0.959	0.923	0.978
**Pneumonia**	Lip	Overall*	46	0.980	0.964	0.989
		Meas1	25	0.987	0.971	0.994
		Meas3	17	0.887	0.728	0.955
	Skin fold	Overall	85	0.963	0.944	0.975
		Meas1	35	0.953	0.911	0.975
		Meas3	7	1.000		
		Meas4	8	1.000		
		Meas5	35	0.959	0.923	0.978
**Controls**	Lip	Overall	20	0.958	0.900	0.982
		Meas1	8	0.939	0.768	0.985
		Meas2	12	0.977	0.929	0.993
	Skin fold	Overall	21	0.948	0.880	0.978
		Meas1	8	0.961	0.846	0.991
		Meas2	13	0.937	0.820	0.979

** The data from measurers with ≤ 2 measurement sessions are not presented separately but are included in the "Overall" results.*
